# Automatic analysis framework based on 3D-CT multi-scale features for accurate prediction of Ki67 expression levels in substantial renal cell carcinoma

**DOI:** 10.1186/s13244-023-01465-y

**Published:** 2023-07-19

**Authors:** Huancheng Yang, Jiashan Lin, Hanlin Liu, Jiehua Yao, Qianyu Lin, Jiaxin Wang, Feiye Jiang, Liying Wei, Chongyang Lin, Kai Wu, Song Wu

**Affiliations:** 1 Luohu Clinical Institute, Shantou University Medical College, Shantou, 515000 China; 2 Shenzhen Following Precision Medical Research Institute, Luohu Hospital Group, Shenzhen, 51800 China; 3 Shantou University Medical College, Shantou University, Shantou, 515000 China; 4 Department of Urology, Peking University Shenzhen Hospital, Shenzhen, 518036 China; 5 Department of Radiology, The Third Affiliated Hospital of Shenzhen University (Luohu Hospital Group), Shenzhen, 518000 China; 6 Department of Urology, South China Hospital, Health Science Center, Shenzhen University, Shenzhen, 518116 China

**Keywords:** Renal cell carcinoma, Ki67, Multi-scale features

## Abstract

**Purpose:**

To investigate the effectiveness of an automatic analysis framework based on 3D-CT multi-scale features in predicting Ki67 expression levels in substantial renal cell carcinoma (RCC).

**Methods:**

This retrospective study was conducted using multi-center cohorts consisting of 588 participants with pathologically confirmed RCC. The participants were divided into an internal training set (*n* = 485) and an external testing set (*n* = 103) from four and one local hospitals, respectively. The proposed automatic analytic framework comprised a 3D kidney and tumor segmentation model constructed by 3D UNet, a 3D-CT multi-scale features extractor based on the renal–tumor feature, and a low or high Ki67 prediction classifier using XGBoost. The framework was validated using a fivefold cross-validation strategy. The Shapley additive explanation (SHAP) method was used to determine the contribution of each feature.

**Results:**

In the prediction of low or high Ki67, the combination of renal and tumor features achieved better performance than any single features. Internal validation using a fivefold cross-validation strategy yielded AUROC values of 0.75 ± 0.1, 0.75 ± 0.1, 0.83 ± 0.1, 0.77 ± 0.1, and 0.87 ± 0.1, respectively. The optimal model achieved an AUROC of 0.87 ± 0.1 and 0.82 ± 0.1 for low vs. high Ki67 prediction in the internal validation and external testing sets, respectively. Notably, the tumor first-order-10P was identified as the most influential feature in the model decision.

**Conclusions:**

Our study suggests that the proposed automatic analysis framework based on 3D-CT multi-scale features has great potential for accurately predicting Ki67 expression levels in substantial RCC.

**Critical relevance statement:**

Automatic analysis framework based on 3D-CT multi-scale features provides reliable predictions for Ki67 expression levels in substantial RCC, indicating the potential usage of clinical applications.

**Graphical abstract:**

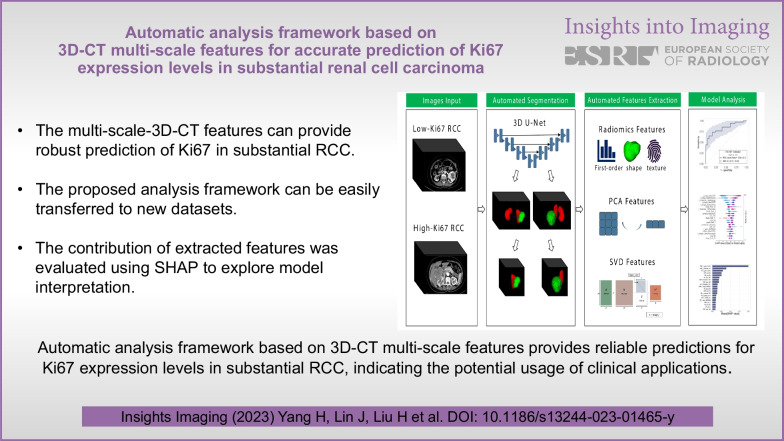

**Supplementary Information:**

The online version contains supplementary material available at 10.1186/s13244-023-01465-y.

## Key points

- The multi-scale 3D-CT features can provide robust prediction of Ki67 in RCC.
- The proposed analysis framework can be easily transferred to new datasets.
- The contribution of extracted features was evaluated using SHAP to explore model interpretation.

**Supplementary Information:**

The online version contains supplementary material available at 10.1186/s13244-023-01465-y.

## Introduction

Renal cell carcinoma (RCC) is the most common renal tumor in adults and the second most common urinary tract tumor, accounting for 3% of all cancers [1]. Over the last twenty years, there has been a consistent annual rise of 2% in the incidence of RCC worldwide [2]. At initial diagnosis, a large percentage (approximately 20–30%) of RCC patients have distant metastases, and a substantial number (approximately 20–30%) of patients with localized RCC develop metastases even after curative nephrectomy, with 10–15% of cases ultimately resulting in fatality [3]. As a result, it is crucial to accurately identify RCC patients with poor prognoses at an early stage, which holds significant clinical significance.

Ki-67 is a nuclear antigen that reflects cell proliferation status, expressed throughout the cell cycle of proliferating cells except for G0 phase, and is closely associated with tumor proliferation and invasion. Many studies have suggested that Ki-67 is a useful prognostic marker in RCC, with high expression levels being associated with poor prognosis and advanced clinicopathological features [4–6]. Detection of Ki67 requires a pathological puncture, which is an invasive procedure that carries risks such as tumor needle tract metastasis or infection. Furthermore, due to the possibility of RCC patients experiencing recurrence or multiple repeated surgeries, the development of a noninvasive and dynamic predictive model for Ki67 holds significant clinical value.

CT is the predominant imaging modality for preoperative assessment and postoperative surveillance of RCC patients, and it represents a critical component of standard patient care [7]. In recent years, machine learning algorithms have shown promise in the field of medical image analysis and have demonstrated their ability to accurately predict Ki67 at many cancers [8–11]. Despite their promising predictive performance, many of these models are limited in terms of their practical applicability in clinical settings. Two specific limitations include the reliance on manual annotation by radiologists for target regions learned by the models, which is impractical for clinical practice. Additionally, most of the models only offer classification results without providing insight into the decision-making logic behind those results, leading to distrust and hesitation among clinicians in implementing them [12].

To the best of our knowledge, there was currently no literature available that reports the predictive value of machine learning-based CT features for Ki67 in substantial RCC. Given the complexity and diversity of analysis, it is crucial to extract more comprehensive image features to enable accurate prediction. In this study, we propose an automatic analysis framework that includes three key modules: a 3D kidney and tumor segmentation model constructed using 3D UNet, a 3D-CT multi-scale features extractor based on the renal–tumor and a low or high Ki67 prediction classifier using XGBoost. To ensure a robust framework, we employed a fivefold cross-validation strategy. Additionally, we used a quantitative model interpretation method called SHAP to explore the contribution of each feature.

## Materials and methods

### Study population

This study is a retrospective analysis of multi-center datasets, encompassing 588 participants who underwent nephrectomy for substantial renal cell carcinoma from 2017 to 2022 in five medical centers. The dataset was divided into an internal group of 485 participants and an external testing group of 103 participants, with the internal group further divided into a training group of 388 participants (80%) and an internal validation group of 97 participants (20%). The protocols for collecting the data were approved by the local institutional review board (KY2022-036-01) and informed consent was waived, as the study relied on anonymous clinical data and images.

**Fig. 1:**
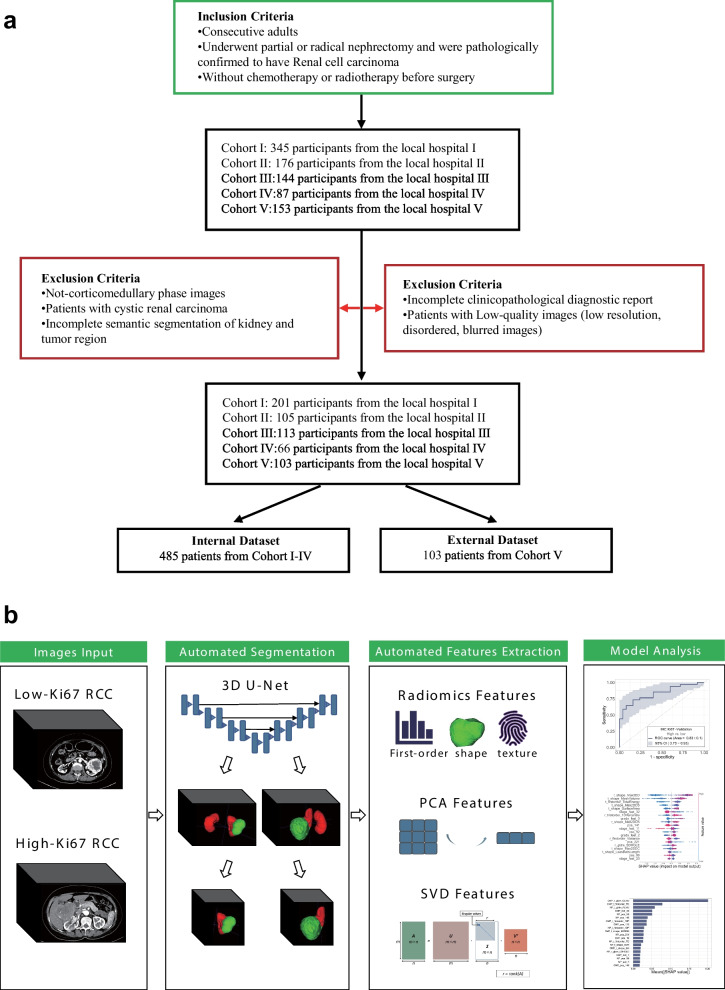
Analysis framework based on 3D-CT multi-scale features for accurate prediction of Ki67 expression levels in substantial renal cell carcinoma. **a** Flowchart of the procedures for the study. **b** Automatic analysis framework: A 3D UNet model is utilized to segment lesions and extracted features from multi-scale are used for prediction

In adherence to the inclusion criteria and processing protocols outlined in Fig. 1a, we disregarded cases with inadequate clinical and pathological information and limited our analysis to corticomedullary phase images. Inclusion Criteria: 1. Underwent partial or radical nephrectomy and were pathologically confirmed to have substantial renal cell carcinoma; 2. Consecutive adults; and 3. Without chemotherapy or radiotherapy before surgery. Exclusion Criteria: 1. Incomplete semantic segmentation of kidney and tumor region; 2. Patients with cystic renal carcinoma; 3. Not-corticomedullary phase images; 4. Incomplete clinicopathological diagnostic report; and 5. Patients with low-quality images (low resolution, disordered, and blurred images).

### Images preparation and segmentation

Following a thorough selection process, the images were meticulously annotated and divided into kidneys and kidney cancer segments by a team of two experienced radiologists and two adept medical students. Based on the 3D UNet network [13], we constructed an automated kidneys and kidney cancer segmentation model. The principle behind this was to identify a function "g" consisting of a set of heuristics that adjusts normalization and resampling techniques. Hyperparameters, including pooling operations, batch size, and patch size, were chosen based on the characteristics of the dataset. To guarantee precision, the segmentation results were reviewed and revised by a specialist with over 20 years of experience and multiple observers. This process was repeated to ensure accuracy in the delineation and prepare for further model training.

### Multi-scale features extractor

The 3D-CT multi-scale features extractor comprised a radiomics feature extractor, a PCA (principal component analysis) matrix dimensionality reduction feature extractor and an SVD (singular value decomposition) matrix decomposition feature extractor. The PyPi pyradiomics [14] module was utilized to extract the texture, morphological and statistical features of the CT images, yielding 100 features for each of the ROI. Subsequently, a dimensional reduction was performed on the segmented voxels of the CT images, with adjustments made to image resolution, normalization using mean and standard deviation values, cropping to include only the kidneys or tumor regions, and filling of empty regions with minimal pixels (see the supplementary file for details and Additional file 1: Fig. S1). Three hundred and twenty features by dimensionality reduction, which represents the original voxel information, was performed by PCA (256 features for each of the ROI) and SVD (64 features for each of the ROI).

### Model construction and explaining

Gradient boosting decision trees (XGBoost, v1.3.3) [15] were utilized to predict the Ki67 expression levels. A combination of Ki67 levels greater than or equal to 5% was classified as high risk and Ki67 levels less than 5% as low risk. To evaluate the performance of single renal features, single tumor features, and combined renal–tumor features, three classifiers were constructed for Ki67 (high risk vs. low risk).

The process of decision-making was explored with the aid of SHapley Additive exPlanations (SHAP) [16] by decomposing the model's decision into individual feature influences. A high SHAP value indicates a significant impact on the model's decision. The accuracy and area under the receiver operating characteristic curve (AUROC) were quantified with a 95% confidence interval and were deemed statistically significant if the p-value was less than 0.05. The statistical analysis was performed in Python (v3.8) and R (v3.6.3).

## Results

### Participant information

A cohort of 588 individuals diagnosed with substantial RCC, comprising 298 (236, 62) males and 290 (149, 41) females, participated in the study. A summary of their basic and clinical information is presented in Table 1. The internal set of 485 participants was randomly divided into a training set, consisting of 388 cases (80%), and a validation set, comprised of 97 cases (20%). The remaining 103 participants were assigned to an external testing set (Fig. 1a). The automatic analysis framework is illustrated in Fig. 1b.

**Table 1:** Basic, clinical and pathologic characteristics of patients involved in this research

Characteristic	Internal dataset	External dataset
Participants (588)	485	103
Age (year)	51.60 ± 14.99	53.30 ± 13.11
Sex		
Female	249	41
Male	236	62
Histologic subtype		
Clear cell renal cell carcinoma	284	86
Chromophobe renal cell carcinoma	91	7
Papillary renal cell carcinoma	69	6
Other types	41	4
Pathologic tumor stage		
Low stage (T1/T2)	363	65
High stage (T3/T4)	92	38
Not available	30	0
Pathologic tumor grade		
Low grade (G1/G2)	257	52
High grade (G3/G4)	96	40
Not available	132	11
Immunohistochemistry (Ki67)		
Low Ki67 (< 5%)	297	56
High Ki67 (> = 5%)	188	47
Not available	0	0

### Multi-scale features framework provides robust analysis capability

**Fig. 2:**
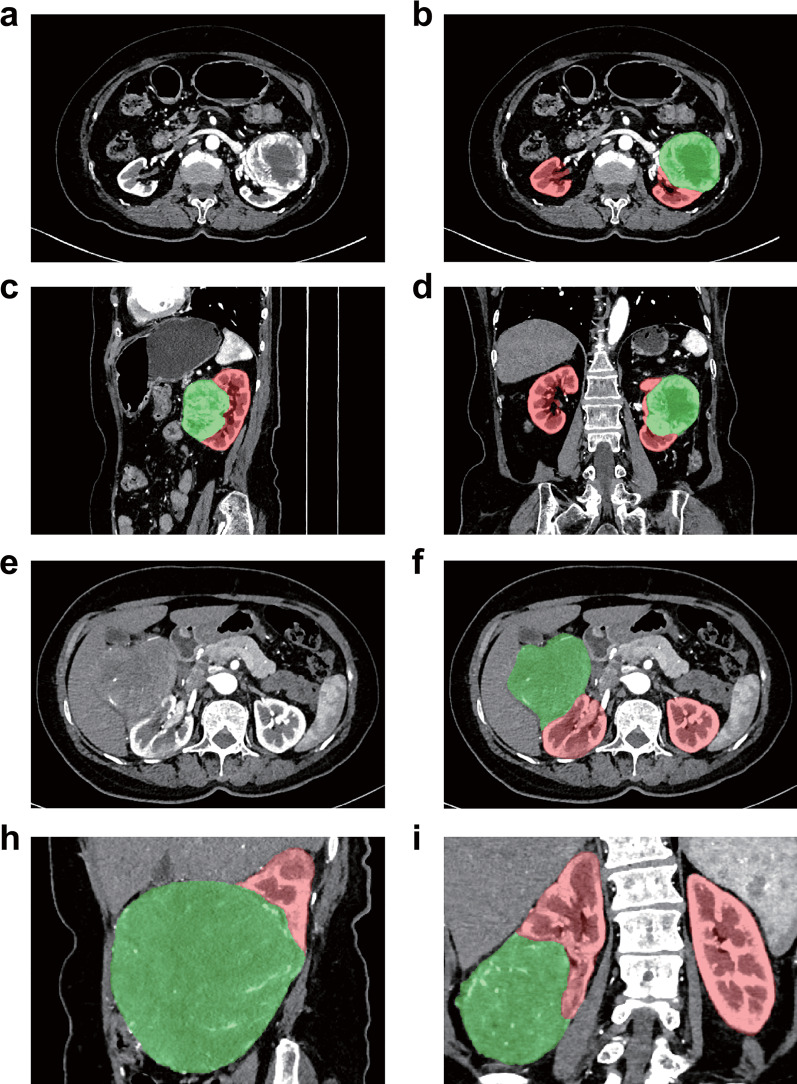
CT images after automatic segmentation by 3D UNET in external dataset. **a**–**d** Male, 77, clean cell renal cell carcinoma, underwent radical nephrectomy, high Ki67 (10%). **e–i** Male, 60, chromophobe renal cell carcinoma, underwent radical nephrectomy, low Ki67 (3%)

The 3D region of the kidney and tumor were expertly segmented from CT images through a structure-based 3D UNet. In Fig. 2, the segmentation model demonstrates outstanding performance in test cases, with the red ROI symbolizing the kidney and the green ROI representing the tumor.

**Fig. 3:**
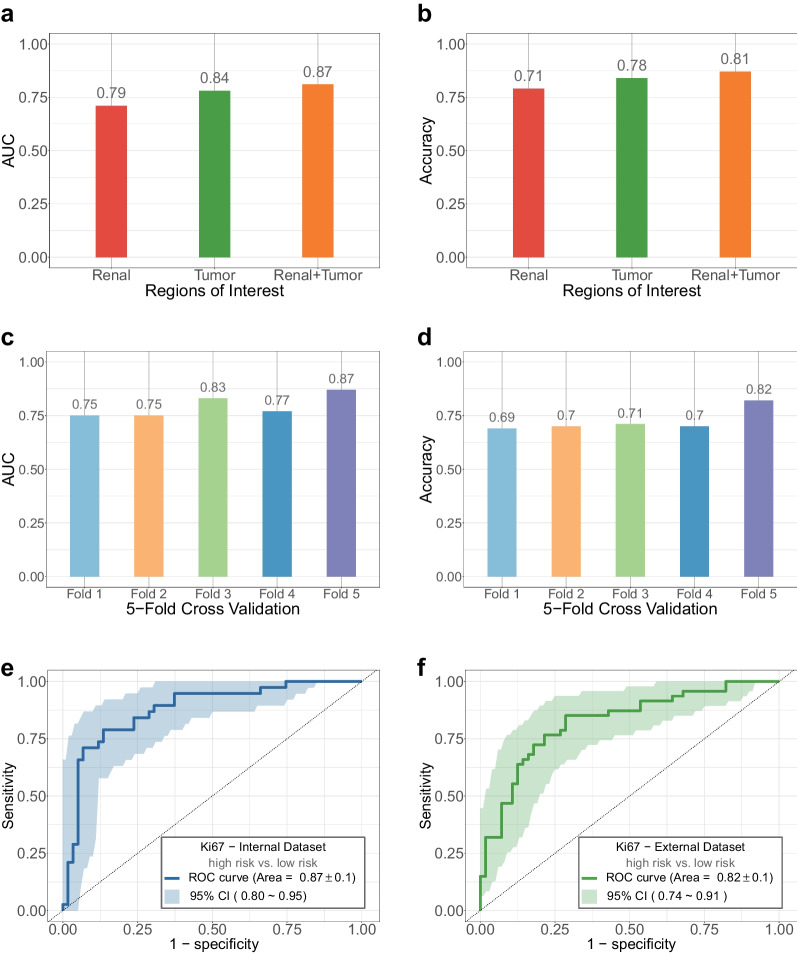
Analysis and results of multi-scale features extraction and performance of proposed framework. **a**, **b** The AUROC and accuracy of different region feature extraction. **c**, **d** The AUROC and accuracy of Ki67 expression levels prediction model in internal validation set by the fivefold cross-validation. **e**, **f** The AUROC of Ki67 expression levels prediction model in internal dataset and external dataset

The AUROC from single renal features, single tumor features, and renal–tumor features, as shown in Fig. 3a, b, was 0.79 ± 0.1, 0.84 ± 0.1, and 0.87 ± 0.1 for the classification of Ki67, with an accuracy of 0.71, 0.78 and 0.81 at a 95% confidence level, respectively. For the internal validation, the AUROC of the low vs. high Ki67 prediction was 0.75 ± 0.1, 0.75 ± 0.1, 0.83 ± 0.1, 0.77 ± 0.1 and 0.87 ± 0.1, with an accuracy of 0.67, 0.70, 0.71, 0.70 and 0.82 by the fivefold cross-validation, respectively (Fig. 3c, d). The AUROC from the optimal model was 0.87 ± 0.1 and 0.82 ± 0.1 for low vs. high Ki67 prediction at the internal validation set (Fig. 3e) and external testing set (Fig. 3f), respectively.

### Feature contribution evaluation by SHAP values

**Fig. 4:**
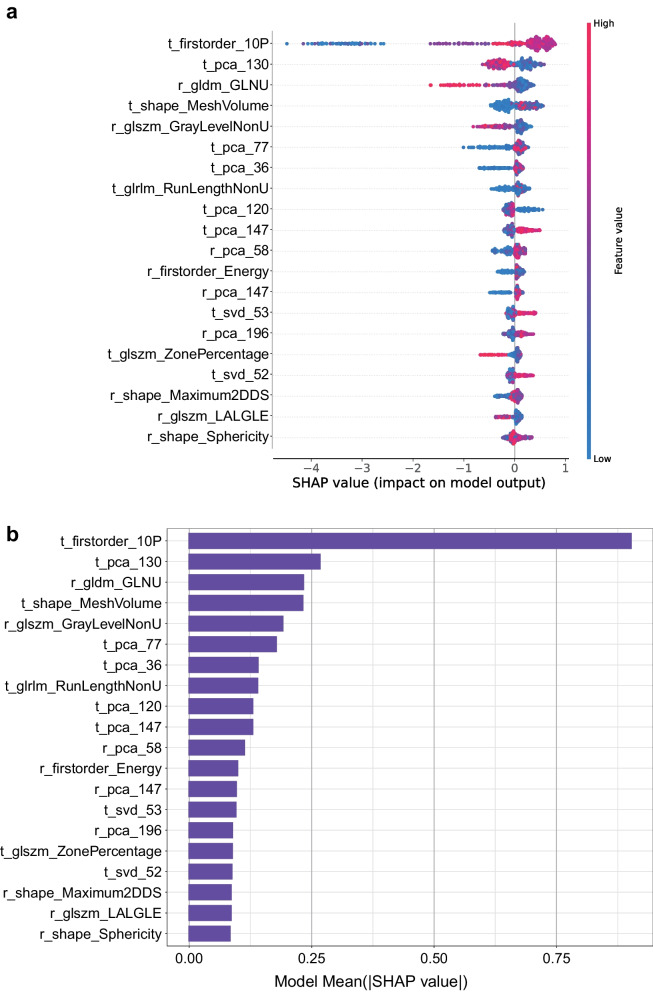
Ranking of SHAP values for the explanation of Ki67 expression levels prediction model. **a**, **b** Barplot and bee-swarm plot display the SHapley Additive exPlanations (SHAP) values for the training set of Ki67 expression levels prediction model

The contribution of the imaging features to the model's prediction was assessed by computing the SHAP values, which decomposed the decision of the model into the influence of individual features for each sample. The top-20 driver features were visualized in bee-swarm-plots (Fig. 4a) and bar-plots (Fig. 4b) for low vs. high Ki67 prediction. The bee-swarm plot depicts the SHAP values and feature values across the original dataset, with redder dots indicating larger eigenvalues and bluer dots indicating lower eigenvalues, and positive SHAP values signifying a higher likelihood for the corresponding prediction.

In Fig. 4a, b, the tumor first-order 10P and pca 130 were found to be critical in low vs. high Ki67 prediction. To test the noise reduction capability of our model, we conducted a feature elimination experiment by removing features with SHAP values equal to zero and re-training the original model. The results indicated that the model's performance remained comparable, and the AUROC achieved 0.87 at a 95% confidence interval.

## Discussion

Despite significant advancements in the diagnosis and treatment of RCC, the overall prognosis still remains dismal [3, 17]. Excessive cell proliferation is a hallmark of cancer. Ki-67, a nuclear protein, serves as a critical marker for assessing tumor proliferation status, and its expression level has significant implications for tumor biology, treatment response, and patient prognosis. At present, pathology is the gold standard for determining tumor Ki-67 expression [4–6]. However, biopsy procedures are invasive and pose risks for dissemination, and are generally not recommended. In clinical practice, noninvasive diagnostic criteria for RCC include imaging signs of corticomedullary phase enhancement and contouring in the nephrographic phase on CT or MRI scans. If Ki-67 expression in RCC can be assessed using imaging modalities, it would offer valuable information to clinicians for making individualized treatment decisions, which is paramount for patient prognosis.

Medical imaging is commonly acknowledged as a product of genetic and molecular-level processes [18, 19]. Consequently, the implementation of artificial intelligence techniques to extract feature from medical images can shed light on the molecular and genotypic foundation of tissues to some degree [20–22]. Many researchers have endeavored to investigate the relationship between CT features and Ki-67 expression level in cancers. The studies of Wu et al. showed that CT texture analysis based on machine learning might be a credible quantitative strategy to predict the Ki67 expression level in hepatocellular carcinoma [9]. Gu et al. found that a CT-based radiomics model could predict a high Ki67 expression level of non-small cell lung cancer [11]. In gliomas, CT features have been found to exhibit a significant correlation with the Ki-67 index [8]. However, the value of features of CT images based on machine learning for predicting the Ki67 expression level of RCC remains uncertain.

In this study, we built a comprehensive machine learning-based approach that includes image processing, semantic segmentation, multi-scale features extraction, and Ki-67 expression level prediction to provide a fully automated analysis framework. Furthermore, to enhance the decision-making ability of the visualization model, we had quantified the impact of each multi-scale features on the model decision using SHAP values.

As known, an excessive number of features may result in overfitting in machine learning. We found that only some features made a decisive impact on the decision-making process of the XGBoost model. As shown in Fig. 4a, b, the tumor first-order 10P were found to be critical in low vs. high Ki67 prediction of RCC, likely reflecting significant differences in growth and physical properties between them. As best as our knowledge, the XGBoost algorithm will assign different weights to each feature and perform the feature selection automatically. However, we are still concerned that the absence of feature selection may lead to overfitting problems. To verify that we conducted a feature elimination experiment by removing features with SHAP values equal to zero and re-training the original model. From this result that the AUROC still achieved 0.87, number of features has a small effect on overfitting when using the XGBoost algorithm for model training.

Indeed, a reliable model must not only be able to adapt to any given dataset in real-world scenarios, but also produce consistent and stable results [23]. In our study, we utilized an automatic segmentation-based approach to delineate the kidney and tumor region. This not only significantly reduces the time and cost involved, but also ensures reproducibility for research result while possessing a certain level of generalizability.

Furthermore, our multi-scale features extraction strategy can be seamlessly applied to novel datasets for diverse tasks. Moreover, we employed a fivefold cross-validation approach and a heterogeneous dataset for model validation. Despite the potential risk of overfitting, our model's external test AUROC still achieved an impressive value of 0.84.

Although this study has demonstrated promising results in predicting Ki67 expression levels in substantial RCC patients, there are several limitations that should be addressed in future studies. Firstly, the retrospective and multi-center nature of this study may lead to data heterogeneity and overfitting, which can affect the model's performance. Secondly, while the model can predict low and high Ki67 expression levels, the impact of this prediction on patient outcomes remains unknown. Long-term follow-up studies and prospective studies are necessary to evaluate the clinical significance of Ki67 expression levels predicted by the model. Finally, further research is needed to optimize and improve the accuracy of the model, potentially through the incorporation of additional imaging features or the use of deep learning algorithms.

In conclusion, our study suggests that the proposed automatic analysis framework is capable of predicting the Ki67 expression levels in substantial RCC patients automatically, noninvasively, and dynamically. This prediction can serve as a valuable reference for clinical treatment decisions.

## Supplementary Information

**Additional file 1.** Supplementary Method. **Fig. S1**. The multi-scale features extractor. 200 Radiomics features were integrated with 512 PCA and 128 SVD features as the 840 combined features to the classifier.

## Data Availability

The original images and data used in this study are available from the corresponding author by request.

## Abbreviations

AUROC: Area under the receiver operating characteristic curve
RCC: Renal cell carcinoma
ROC: Receiver operating characteristic
ROI: Regions of interest
SHAP: The Shapley additive explanations
vs.: Versus
