# Supplementary material for: Automatic analysis framework based on 3D-CT multi-scale features for accurate prediction of Ki67 expression levels in substantial renal cell carcinoma
Source: Insights Imaging. 2023 Jul 19;14:130. doi: 10.1186/s13244-023-01465-y (PMC10356689; doi:10.1186/s13244-023-01465-y)
Supplement: Supplementary file 1 — Additional file 1. Supplementary Method. Fig. S1. The multi-scale features extractor. 200 Radiomics features were integrated with 512 PCA and 128 SVD features as the 840 combined features to the classifier. [file 13244_2023_1465_MOESM1_ESM.pdf]

Automatic analysis framework based on 3D-CT multi-scale features for accurate prediction of Ki67 expression levels in substantial renal cell carcinoma

ELECTRONIC SUPPLEMENTARY MATERIAL

Supplementary Figure

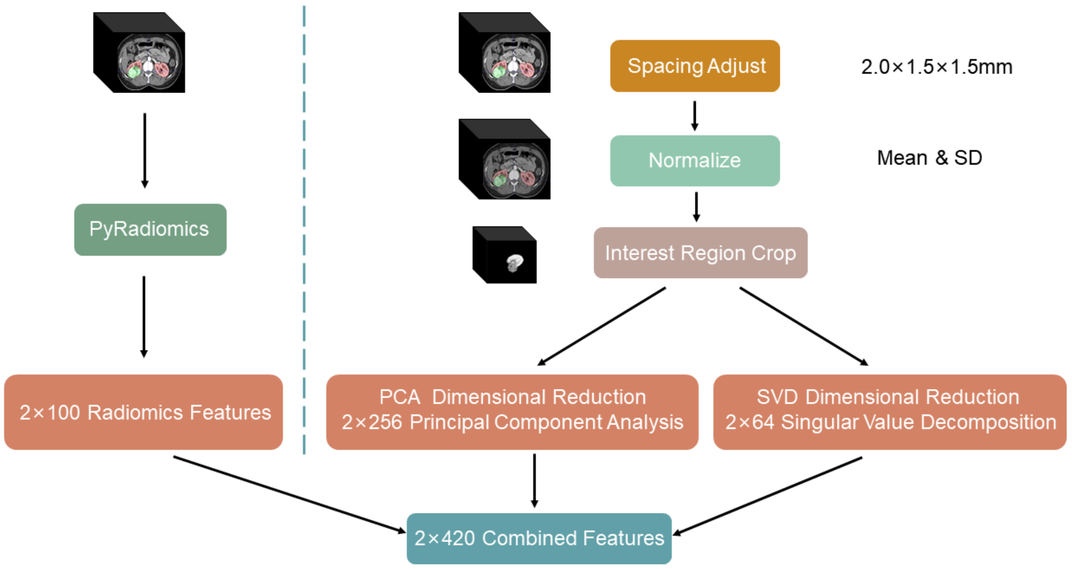

Fig.1 The multi-scale features extractor. 200 Radiomics features were integrated with 512 PCA and 128 SVD features as the 840 combined features to the classifier.

Supplementary Method

The 3D-CT multi-scale features extractor comprised a radiomics feature extractor, a PCA (Principal Component Analysis) matrix dimensionality reduction feature extractor, and an SVD (Singular Value Decomposition). matrix decomposition feature extractor. Pyradiomics module in PyPi was utilized to extract the texture, morphological and statistical features of the raw CT images, yielding 100 features for each of the ROI.

In addition, we performed dimension reduction on the segmented voxels of CT images. Firstly, we adjusted the resolution of all the raw images (ranged from 0.5mm to 2.5mm) into a uniform value 2.0*1.5*1.5mm by using the interpolation method from scikit-image (v 0.20). Secondly, we calculated the mean value, standard deviation (std) value, the 0.5% and 99.5% interval of all the ROI (tumor and kidney regions) from the training set. Thirdly, the voxels outside the 0.5%-99.5% interval were clipped to the interval edges. Fourthly, we performed image normalization based on the mean and std value as expressed in the following formula. Fifthly, the target region was cropped and empty regions of them were filled with the minimal value. Lastly, singular value decomposition (SVD) and principal component analysis (PCA) were used to reduce the dimension of the cropped region into 128 and 512 by scikit-learn tools. To summarize, a total of 840 features (radiomics (200), SVD (128), and PCA (512) was extracted for each phase of image (Fig.S1).

$$Normalize(Voxels) = \frac{Voxels - Mean_{ROIs\ of\ all\ training\ set}}{Std_{ROIs\ of\ all\ training\ set}}$$
